# Investigation on Cutting Performance of Micro-Textured Cutting Tools

**DOI:** 10.3390/mi10060352

**Published:** 2019-05-28

**Authors:** Qinghua Li, Chen Pan, Yuxin Jiao, Kaixing Hu

**Affiliations:** College of Machinery and Vehicle Engineering, Changchun University, Changchun 130022, China; 17743125369@163.com (C.P.); jiaoyx1025@163.com (Y.J.); 13756947307@163.com (K.H.)

**Keywords:** micro hole, cutting performance, cutting force, tool wear, surface roughness

## Abstract

This paper explores the influence of micro textures on cutting performance of polycrystalline cubic boron nitride (PCBN) tools from two aspects, that is, tool wear and machined surface roughness. By designing micro-hole textures with different forms and scales on the rake face of tools when PCBN tools turn hardened steel GCr15, and combining finite element analysis (FEA) technology and cutting experiments, the cutting performance of micro-textured tools is simulated and analyzed. This paper analyses the influence of micro textures on tool wear and machined surface roughness by analyzing cutting force, Mises stress and maximum shear stress of tool surface. Results of finite element analysis (FEA) and cutting experiments show that the reasonable micro-hole textures can significantly alleviate tool wear and improve machined surface quality when compared with the non-textured tools. Besides, the size of micro-hole textures on the rake face play an important role in reducing the cutting force and tool wear. This is mainly because micro-hole textures can reduce cutting force and improve tool surface stress. Finally, by designing reasonable micro-hole textures on the rake face, the problems of bad roughness of machined workpiece and severe tool wear of PCBN tools in cutting GCr15 material are solved. Consequently, the paper shows that micro-hole textures have a positive effect on improving the cutting performance of tool.

## 1. Introduction

As for studying surface micro textures, many scholars [1,2,3,4] first applied micro textures to the motion pairs which contacted each other and produced friction relative motion, and studied the effect of micro textures on friction performance. The results showed that micro textures could reduce friction between moving components, and reduce friction coefficient, and improve wear resistance of components. Moreover, micro textures had the function of storing lubricant and could better improve friction conditions between components. Yin et al. [5,6] used fluid dynamics (CFD) technology and found that the bearing capacity of sliding bearings was related to the distribution of micro textures in the bearing position, and reasonable micro textures design could effectively improve the performance of sliding bearings. Zhang et al. [7] found that sinusoidal micro textures had better friction reduction performance than ordinary linear micro textures. Samanta et al. [8] found that the shear strength of sand steel in contact with each other was closely related to the type of micro textures.

It is a new direction to apply micro textures in the field of manufacture. Micro textures provides a new method to study the cutting force and tool wear produced in the cutting process. It is important that micro textures has achieved very good results in reducing cutting force, delaying tool wear and improving the quality of cutting surface [9,10,11,12,13,14]. Yang et al. [15] found that reasonable micro textures of tool surface could effectively reduce friction coefficient, reduce cutting force and improve the surface quality of the workpiece. Liu et al. [16] revealed that micro textures reduced the contact area between ceramic tool and workpiece, and increased the heat dissipation area, and then increased the heat exchange between air and friction surface, which were conducted to reducing cutting temperature. Qi et al. [17] had proven that micro grooves could improve the friction between the rake face and chips of cemented carbide tools, and reduce the cutting temperature and the cutting force, on this account, micro grooves effectively improved the bonding phenomenon of titanium alloys and the heat transfer ability of tools, and improved the wear resistance of tools. Long et al. [18] illustrated that the circular arc micro textures of carbide tools could significantly reduce the main cutting force, the radial thrust force and the axial thrust force, as well as the cutting temperature.

Lei et al. [19] confirmed that micro-textured tools with lubricant had a positive effect on reducing the main cutting force, the radial thrust force and the axial thrust force when cutting low carbon steel under lubrication conditions. Sugihara et al. [20] revealed the restraining effect of micro textures on crescent depression wear of cemented carbide tool and the relationship between micro-textured size and fatigue resistance. Sugihara and Enomoto [21] argued that the micro textures on the tool surface could significantly improve the viscous properties, and the micro-groove textures parallel to the main cutting edge had the best anti-bonding performance. Obikawa et al. [22,23] concluded that appropriate micro textures on the rake face of cemented carbide tools could reduce the cutting force and change the flow direction of chips when cutting Ti-6Al-4V. Ling et al. [24] deemed that the surface texture could effectually reduce the adhesion force between the tool surface and chips during drilling, and reduce the tool bond wear and improve the tool life.

At present, the method of finite element analysis (FEA) is widely used in the field of cutting research. Xu et al. [25] applied FEA to simulate the cutting performance of polycrystalline diamond (PCD) micro-textured tools. Kandr et al. [26] used FEA method to analyze the influence of tool geometry angle and cutting parameters on cutting force in processing titanium alloy. Ulutan and Turul [27] also used FEA to study tool wear in cutting titanium alloy. The FEA method not only reduces the number of cutting tests, but also makes a reasonable analysis and exploration of cutting process in theory. Therefore, it is necessary that study the influence of micro-hole textures on the cutting force and tool wear and its mechanism by using FEA method theoretically.

However, prior studies on the micro textures of tool surface had mostly focused on cemented carbide tools, coated tools and ceramic tools, but seldom applied to polycrystalline cubic boron nitride (PCBN) tools. Therefore, in order to make up for the lack of research on the cutting performance of PCBN tools, this paper would design micro textures with different forms and scales on the rake face. By means of finite element analysis (FEA) and cutting experiments, the influence of micro textures on tool wear of PCBN tools and machined surface roughness of workpiece in cutting GCr15 were studied and investigated.

## 2. Optimizing Cutting Parameters

### 2.1. Design Experiments

The workpiece: hardened steel GCr15 with a diameter of 50 mm and a length of 25 mm. Rockwell hardness of GCr15 was 58HRC.

The cutting tool: PCBN tool, whose brand was FBS9300, supported by FUNIK company in He Nan province of China (Website: http://www.funik.com/product/36-cn.html).

Experiment scheme: because of high hardness of workpiece GCr15, in order to ensure the cutting edge strength, the negative rake angle *r*_0_ = 6°, the rear angle *a*_0_ = 6° and the main deviation angle *k_r_* = 91° were used.

In previous researches on PCBN tools cutting hardened steel [28,29,30], scholars had done a lot of researches on surface quality of machined hardened steel with feed *f*. The range of feed *f* selection was greater than or equal to *f* = 0.1 mm/r. Under condition of constant cutting speed and cutting depth, surface quality of hardened steel with feed *f* = 0.1 mm/r was better. So This paper chose feed *f* = 0.1 mm/r. Additionally, this experiment mainly studied the influence of micro textures on cutting performance of tools, and cutting speed and cutting depth had a great influence on strength of micro textures on tool surface. Therefore, it was necessary to optimize cutting speed and cutting depth, and multiple sets of experiments were designed in Table 1. By designing experiments that non-textured PCBN tools cut hardened steel GCr15, the cutting speeding and cutting depth were optimized in terms of machined surface roughness of workpiece. The main purpose was to make preparations for studying the effect of micro textures on tool wear and roughness of workpiece.

### 2.2. Analysis of Experiment Results

**Figure 1:**
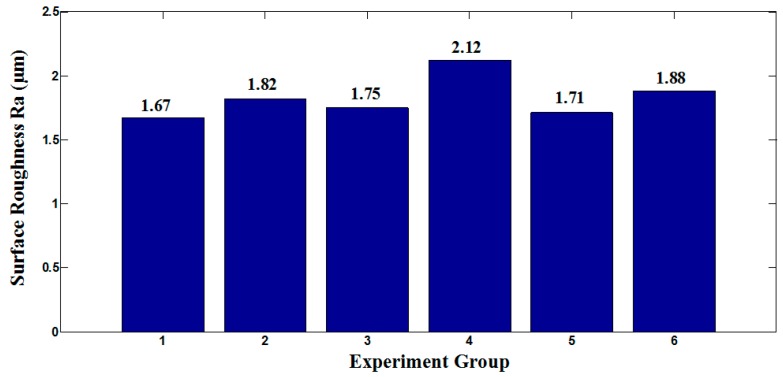
The effect of cutting speed on surface roughness of workpiece.

As shown in Figure 1, the machined surface roughness was made into the bar chart. By analyzing Figure 1, it was found that the surface roughness of group 1 was the smallest, therefore, the machined surface quality was the best. Machined surface roughness of other groups were larger than group 1.

Therefore, group 1 was chosen as the optimum cutting parameter to study the effect of micro textures on cutting performance of tools.

## 3. Finite Element Analysis (FEA)

### 3.1. Finite Element Model of Micro-Hole Tools

**Figure 2:**
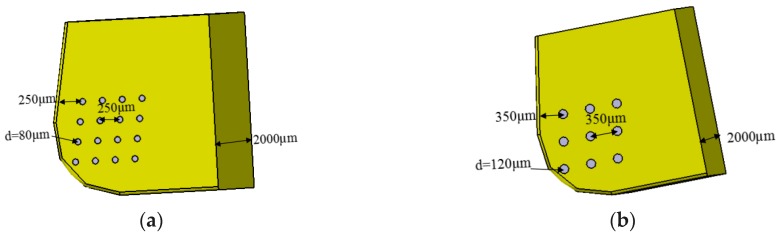
3D model of micro-hole tools: (**a**) *d* = 80 µm micro-hole tool; (**b**) *d* = 120 µm micro-hole tool.

Two different types of micro-hole textures were designed on the tool rake face as shown in Figure 2, it was the three-dimensional models of micro-hole tools. The depth of each micro-hole texture was 5 µm.

**Figure 3:**
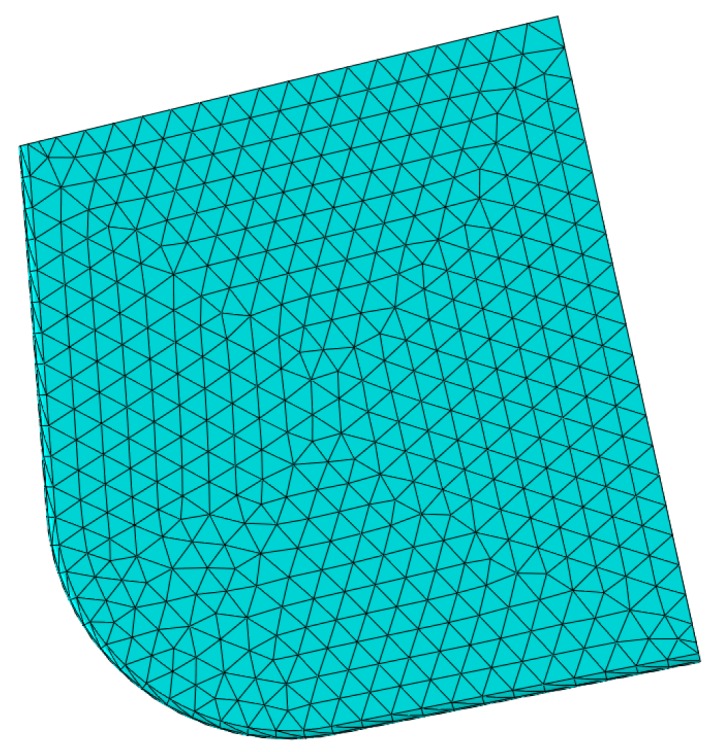
FEA mesh model of cutting tool.

A FEA model was set using the ABAQUS software (Dassault SIMULIA company, Paris, France), and the arbitrary Lagrange-Euler (ALE) adaptive mesh method in ABAQUS software was used to mesh cutting tools, as shown in Figure 3. In boundary conditions of the FEA model, the workpiece was fixed, the cutting tool cloud only move in the feed f direction, and other directions were fixed. 

The workpiece material used in this paper was GCr15, which was a typical plastic material. As for the FEA model of workpiece GCr15, the Johnson-Cook model [31] is a constitutive model and is adopted to describe the constitutive relationship of workpiece material. Because Johnson-Cook constitutive is related to the strain rate, and describes the behavior of the thermal viscoplastic deformation of the material at a high strain rate. Besides, the Johnson-Cook constitutive model shows that the material is changed into strain hardening, strain rate hardening and thermal softening effect at high strain rate. 

Empirical equation of Johnson-Cook constitutive model [31]:

(1) $$\sigma =\left[A+B{\left(\varepsilon \right)}^{n}\right]\cdot \left[1+c\ln\left(\frac{{\dot{\varepsilon }}_{p}}{{\dot{\varepsilon }}_{0}}\right)\right]\cdot \left(1-{\left(\frac{T-T_{0}}{T_{m}-T_{0}}\right)}^{m}\right)$$

where, σ is the flow stress of material; A is yield stress of materials; B is Strain change constant; c, n, and m are material characteristic coefficient; ε is the equivalent plastic strain; ${\dot{\varepsilon }}_{p}$ is the equivalent plastic strain rate; ${\dot{\varepsilon }}_{0}$ is the strain rate reference value; T is the deformation temperature; $T_{0}$ is the room temperature; $T_{m}$ is the melting point temperature of material.

Table 2 is the Johnson-Cook constitutive model parameters of GCr15 [32,33]. Table 3 is mechanical and physical properties of GCr15 [32,33], and Table 4 is the mechanical and physical properties of PCBN tool [32,33].

### 3.2. Analyzing the Effect of Micro-Hole Textures on Tool Wear

In the research, the way to measure tool wear in volume was to select the same point on each rake face of tool, and then measure the wear length of cutting edge and the width width of rake face. By comparing the wear length and width of rake face judged the amount of wear of micro-texture tool. In addition, the wear area of rake face was compared to verify the effect of micro texture on tool wear in more detail.

**Figure 4:**
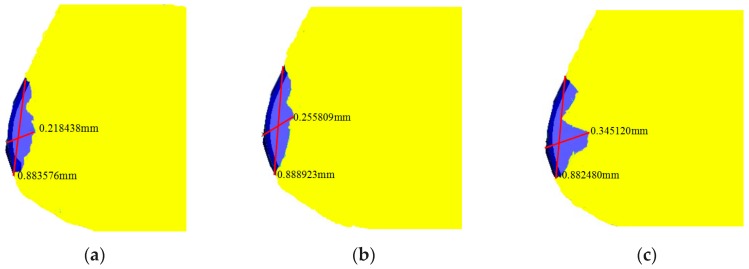
Tool wear: (**a**) *d* = 80 µm micro-hole tool; (**b**) *d* = 120 µm micro-hole tool; (**c**) non-textured tool.

The tool wear diagram was shown in Figure 4 and Table 5. By measuring the wear width of the rake face, it was found that the wear width of rake face of *d* = 80 µm micro-hole tool was the smallest, which was 0.218438 mm. The wear width of the rake face of *d* = 120 µm micro-hole tool was 0.255809 mm. The wear width of non-textured tool rake face reached 0.345120 mm, and wear along the rake face extended obviously longer. In addition, measuring the arc wear length along the cutting edge, the arc wear length of non-textured tool was the shortest, which was 0.882480 mm, followed by the arc wear length of *d* = 80 µm micro-hole tool was 0.883576 mm, and the arc wear length of *d* = 120 µm micro-hole tool was 0.888923 mm, which was the largest wear. Additionally, comparing the wear area of the rake face, it was found that the wear area of *d* = 80 µm micro-hole tool was 0.19300657 mm^2^, whose the wear area was the smallest. The wear area of *d* = 120 µm micro-hole tool was 0.2273945 mm^2^. But the wear area of non-textured tool reached 0.30456152 mm^2^. Obviously, the wear degree of non-textured tool was the most serious. It could be concluded that FEA results showed that the micro-hole textures could effectively reduce tool wear and prolong tool life.

**Figure 5:**
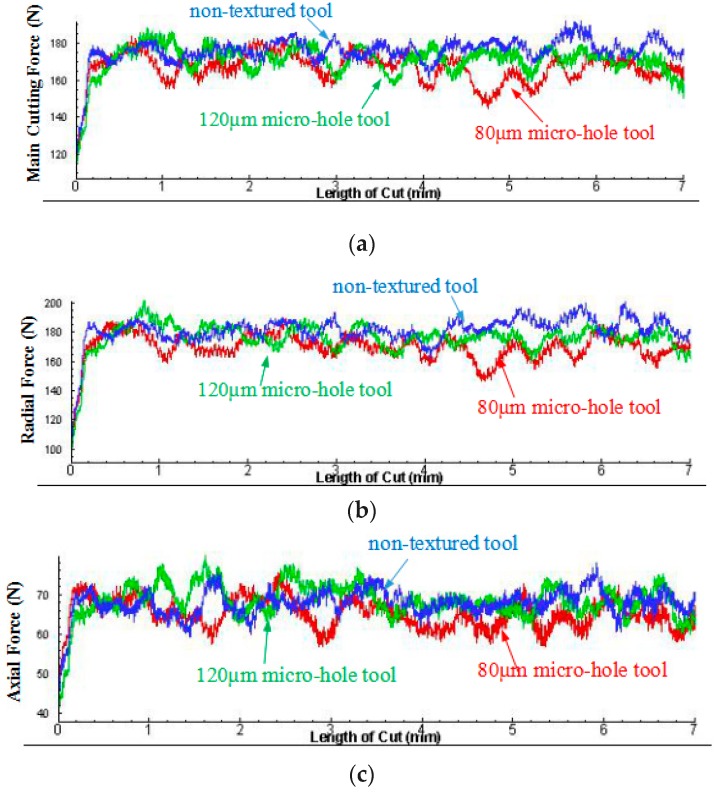
The comparison of cutting force of different PCBN tools: (**a**) the main cutting force; (**b**) the radial force; (**c**) the axial force.

Firstly, the influence of micro-hole textures on tool wear was explained from cutting forces. The comparison of cutting forces of different PCBN tools was shown in Figure 5. The results showed that cutting forces produced by micro-hole tools were smaller than those of the non-textured tools. The main cutting force and the radial force and the axial force produced by *d* = 80 µm micro-hole tool were the smallest. Adversely, cutting forces produced by non-textured tool were largest. Consequently, the smaller cutting force indicated that the micro-hole textures reduced the friction force between the rake face of tools and chips, and the cutting tool was less impacted by forces, which alleviated tool wear. Besides, owing to the different diameters and numbers of micro-hole textures, *d* = 80 µm micro-hole textures were more conducive to reducing the contact area between the rake face and the chip and reducing the friction force. As a result, wear degree of *d* = 80 µm micro-hole tool was less than that of *d* = 120 µm micro-hole tool.

**Figure 6:**
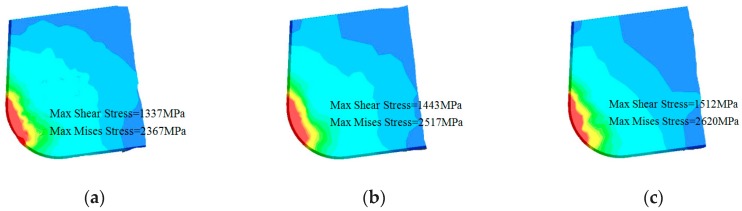
Comparison of max shear stress ans Mises stress: (**a**) *d* = 80 µm micro-hole tool; (**b**) *d* = 120 µm micro-hole tool; (**c**) non-textured tool.

Second, shear stress and Mises stress of cutting tools also explained the influence of micro-hole textures on tool wear. As shown Figure 6, it could be found that the maximum shear stress of *d* = 80 µm micro-hole tool was 1337 MPa, the maximum Mises stress was 2376 MPa. The maximum shear stress of *d* = 120 µm micro-hole tool was 1443 MPa and the maximum Mises stress was 2517 MPa. But the maximum shear stress of non-textured tool was 1512 MPa, the maximum Mises stress was 2620 MPa. The stress state of tool surface could well indicate the fatigue performance of tool, and the smaller stress indicated that the wear degree of tool surface was smaller. Therefore, tool wear of *d* = 80 µm micro-hole tool was the lowest, followed by *d* = 120 µm micro-hole tool.

In conclusion, combined with cutting force and stress of tool surface, this paper confirmed that micro-hole textures could reduce tool wear and prolong tool life, and *d* = 80 µm micro-hole textures were more helpful to alleviate tool wear. At the same time, the relationship between cutting force and tool surface stress and tool wear could be obtained, that was, the smaller the cutting force and tool surface stress, the longer the tool life.

## 4. Cutting Experiment

### 4.1. Fabrication and Machined of Micro Texture on Tool Surface

**Figure 7:**
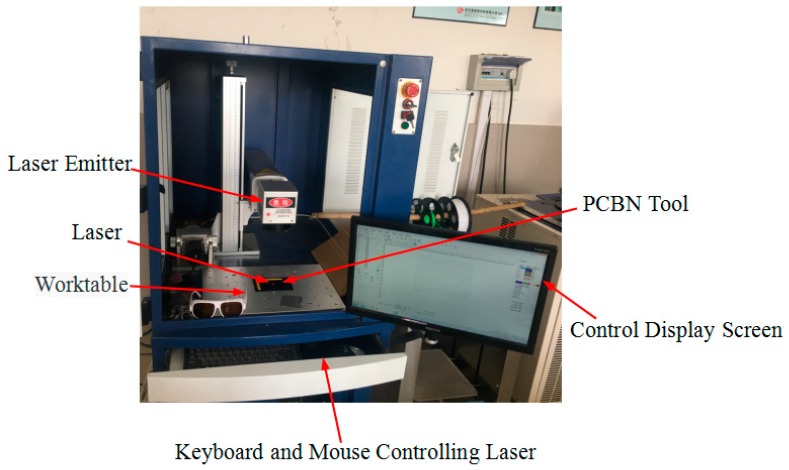
FLY-M10F fully enclosed rotating optical fiber laser marking machine.

**Figure 8:**
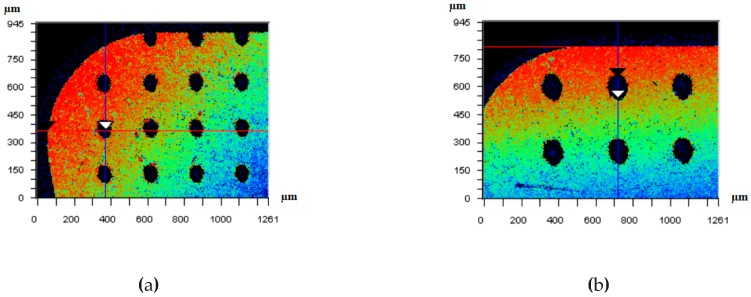
Morphology of micro-hole textures: (**a**) *d* = 80 µm micro-hole; (**b**) *d* = 120 µm micro-hole.

The experiment adopted fully closed with rotating-fiber laser marking machine to fabricate micro-hole textures on the rake face of PCBN tools, as shown in Figure 7. The micro-hole textures on the rake face of PCBN tools were measured by WYKO N7910 optical profiler, as shown in Figure 8.

The cutting experiments were carried out by using micro-hole tools for cutting hardened steel GCr15. In experiments, the cutting parameters and angle of tool was consistent with FEA.

### 4.2. Analyzing Tool Wear of Cutting Experiments

**Figure 9:**
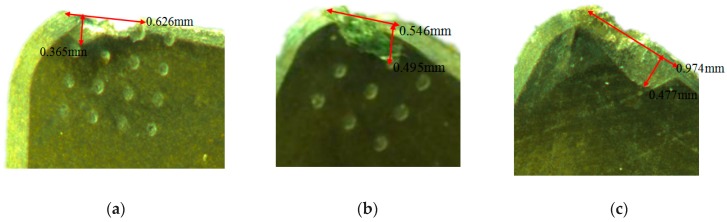
The micrograph of tool wear: (**a**) *d* = 80 µm micro-hole tool; (**b**) *d* = 120 µm micro-hole tool; (**c**) non-textured tool.

In order to clearly compare and analyze tool wear degree, LEICA DVM2500 was used to detect wear degree of tool surface, and analyze the effect of micro-hole textures on tool wear. The micrograph of tool wear was shown in Figure 9.

Compared with tool wear, the wear degree of *d* = 80 µm micro-hole tool was the smallest, wear width of the rake face of *d* = 80 µm micro-hole tool was 0.365 mm, and the arc wear length was 0.626 mm. Wear width of the rake face of *d* = 120 µm micro-hole tool was 0.495 mm, the arc wear length was 0.546 mm. While wear degree of non-textured tool was largest. The tool wear results of cutting experiments basically accorded with FEA.

**Figure 10:**
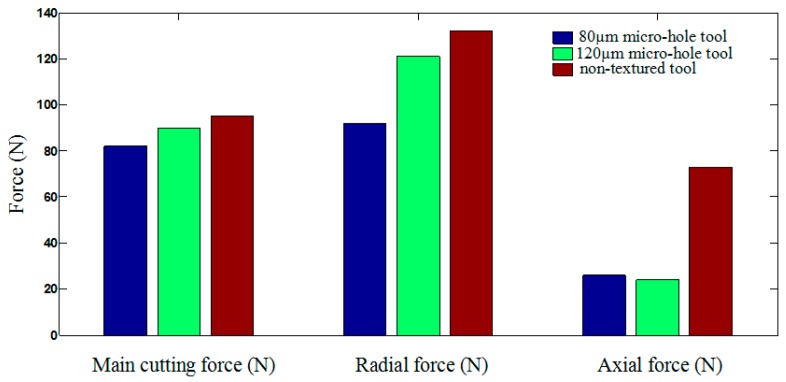
Comparison of cutting forces between micro-hole tool and non-textured tool.

This paper drew the influence curve of different cutting tools on cutting forces and explained reason of tool wear. As shown in Figure 10, it showed that the minimum main cutting force and the minimum radial force were produced by *d* = 80 µm micro-hole tool, followed by *d* = 120 µm micro-hole tool, while the axial force produced by *d* = 80 µm micro-hole tool was slightly larger than that produced by *d* = 120 µm micro-hole tool. However, the main cutting force, the radial force and the axial force produced by non-textured tool were the largest. This also showed that the smaller cutting forces were, the smaller tool wear degree was. The results of cutting experiments had coincided with FEA results, which showed that the micro-hole textures had the effect on reducing tool wear.

### 4.3. Analyzing Machined Surface Roughness of Workpiece

The machined surface roughness of workpiece was also analyzed in order to study the influence of micro-hole textures on cutting performance of cutting tools. Moreover, from the point of view of residual stress state of machined workpiece, the paper explained that micro-hole textures could improve surface quality of workpiece and cutting performance of tools.

**Figure 11:**
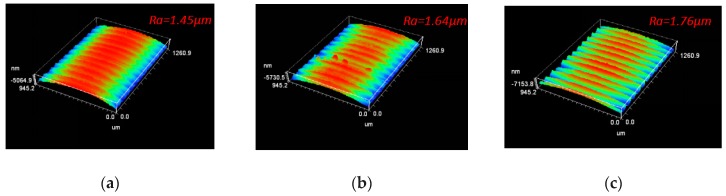
The Surface Roughness Profile of Workpiece: (**a**) *d* = 80 µm micro-hole tool; (**b**) *d* = 120 µm micro-hole tool; (**c**) non-textured tool.

The machined surface roughness of the workpiece machined by different cutting tools was compared and analyzed, as shown in Figure 11. The workpiece surface machined by *d* = 80 µm micro-hole tool was smoothest, the roughness was the smallest and the surface quality of the workpiece was good. The roughness of workpiece surface machined by *d* = 120 µm micro-hole tool was 1.64 µm, surface quality was inferior to that of workpiece machined by *d* = 80 µm micro-hole tool. However, the roughness of workpiece surface machined by non-textured tool was 1.76 µm, whose surface quality was the worst. Firstly, the influence of micro-hole textures on machined surface roughness was explained from cutting forces. By comparing cutting forces mentioned above, it could be found that cutting forces produced by the micro-hole tools were less than those of non-textured tools. The smaller cutting forces indicated that the extrusion effect and shearing effect between rake face of tool and surface material of workpiece was smaller, and that the friction force between tool surface and workpiece surface was also smaller. It was important for micro-hole textures to reduce the roughness of machined surface and achieve better surface quality of workpiece.

**Figure 12:**
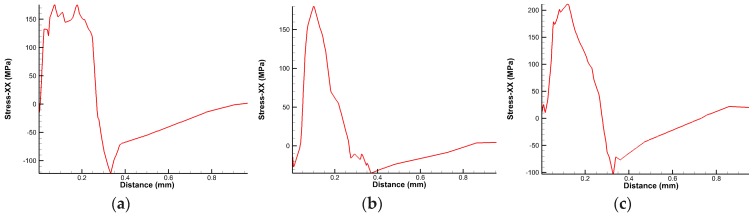
Residual stress of Workpiece Surface: (**a**) *d* = 80 µm micro-hole tool; (**b**) *d* = 120 µm micro-hole tool; (**c**) non-textured tool.

Next, the paper explained the effect of micro-hole textures on workpiece roughness from the residual stress of machined workpiece surface, as shown in Figure 12. By analyzing the residual stress on surface of workpiece, it was found that workpiece surface machined by the micro-hole tools presented compressive stress, while surface of workpiece machined by non-textured tools presented tensile stress, which corresponded to surface roughness of workpiece respectively. It could be seen that the micro-hole textures on tool surface improved the cutting performance of tools, and made workpiece surface show compressive stress, which enhanced fatigue performance of workpiece, and reduced the surface roughness of workpiece, thus micro-hole textures improved the surface quality of workpiece.

## 5. Conclusions

By designing and fabricating micro-hole textures with different sizes and types on rake face of PCBN tools, and combining with FEA and cutting experiments, it was proved that micro-hole textures played an active role in alleviating tool wear and improving machined surface roughness. The following conclusions were drawn:

(1) Compared with non-textured tools, micro-hole textures could effectively prolong tool life, because micro-hole textures reduced cutting forces and stresses of tool surface. Small cutting forces and stresses indicated that tool surface bears less impact force;

(2) Compared with non-textured tools, micro-hole textures could effectively reduce the machined surface roughness, because micro-hole textures reduced cutting force and made workpiece surface show compressive stress. Small cutting forces and compressive stress were beneficial to shape a good surface morphology and improve surface quality of workpiece.

(3) Because micro-hole textures had different forms and sizes, the influence of micro-hole textures on cutting performance of tools was different, *d* = 80 µm micro-hole textures had more significant effect than *d* = 120 µm micro-hole textures in reducing surface roughness of workpiece and alleviating tool wear;

(4) It was found that smaller cutting force was beneficial not only to delay tool wear, but also to improve machined surface quality.

## Figures and Tables

**Table 1:** Cutting parameters of experiments.

Group	Cutting Speed *v* (m/min)	Feed *f* (mm/r)	Cutting Depth *a_p_* (mm)
1	60	0.1	0.2
2	72	0.1	0.2
3	85	0.1	0.2
4	60	0.1	0.3
5	72	0.1	0.3
6	85	0.1	0.3

**Table 2:** GCr15 Johnson-Cook constitutive model parameter [32,33].

*A* (GPa)	*B* (GPa)	*c*	*m*	*n*	*T*_0_	*T_m_*
1.204	1.208	0.036	0.89	0.12	20 °C	1180 °C

**Table 3:** Mechanical and physical properties of GCr15 [32,33].

Material Properties	Young’s Modulus (GPa)	Thermal Conductivity (W/m·K)	Poisson Ratio	Density (g/cm^3^)	Specific Heat (J/kg·°C)
value	210	38	0.3	7.85	480

**Table 4:** Mechanical and physical properties of PCBN [32,33].

Material Properties	Young’s Modulus (GPa)	Thermal Conductivity (W/m·K)	Poisson Ratio	Density (g/cm^3^)	Specific Heat (J/kg·°C)
value	690	120	0.2	3.8	700

**Table 5:** The data of tool wear.

-	The Wear Width *w* (mm)	The Arc Wear Length *L* (mm)	The Wear Area (mm^2^) = *w* × *L*
*d* = 80 µm micro-hole tool	0.218438	0.883576	0.19300657
*d* = 120 µm micro-hole tool	0.255809	0.888923	0.2273945
Non-textured tool	0.345120	0.882480	0.30456152
